# Effect of Early Mobilization on Gait Recovery One Year After Hip Fracture Surgery: A Single-Center Cohort Study

**DOI:** 10.7759/cureus.79133

**Published:** 2025-02-17

**Authors:** Keisuke Nakamura, Tomohiro Sasaki, Takashi Kitagawa, Masayuki Shimizu, Kaoru Aoki

**Affiliations:** 1 Department of Physical Therapy, School of Health Sciences, Shinshu University, Matsumoto, JPN; 2 Department of Rehabilitation, Matsumoto City Hospital, Matsumoto, JPN; 3 Department of Orthopedics, Matsumoto City Hospital, Matusmoto, JPN

**Keywords:** early mobilization, hip fracture, independent walk, older patients, rehabilitation

## Abstract

Purpose: To investigate the effects of early mobilization on walking independence and gait recovery one year after hip fracture surgery.

Materials and methods: This cohort study included 104 patients aged ≥65 years. Patients were divided into two groups: early mobilization (EM; postoperative mobilization on the day after surgery) and late mobilization (LM; postoperative mobilization ≥2 days after surgery) groups. Multivariate logistic regression analysis was performed to investigate the effect of EM on independent walking and recovery to pre-injury walking status one year postoperatively. Independent walking was defined as a walking functional independent measure (FIM) of ≥5.

Results: The number of older patients able to walk independently at discharge and one year postoperatively after hip fracture surgery was 63 (60.6%) and 66 (63.5%), respectively. Multivariate logistic regression analysis revealed that EM was associated with independent walking at one year postoperatively (odds ratio, 3.79; 95% confidence interval, 1.30-11.06; P=0.001). However, EM was not associated with recovery to pre-injury walking status one year postoperatively (P=0.22).

Conclusions: Early postoperative mobilization significantly increased the likelihood of independent walking one year after hip fracture surgery in older adults. Patients who mobilized early were nearly four times more likely to achieve this outcome, underscoring the importance of EM in postoperative care. Further studies are needed to confirm these findings and address barriers to implementing EM in clinical practice.

## Introduction

Hip fractures are a significant concern among older adults due to age-related fragility, with an estimated 16.75 million cases worldwide in 2019 [1]. These fractures often lead to severe mobility impairments, extended rehabilitation, and increased mortality rates in the older population [2-5]. The present study focused on factors that affect recovery and the likelihood of regaining independent ambulation one year after surgery in older hip fracture survivors. Understanding the impact of early postoperative mobilization on long-term functional outcomes is crucial for developing effective rehabilitation strategies for this growing population.

Multiple guidelines recommend early mobilization after hip fracture surgery [6,7], as mobilization is associated with reduced risks of pneumonia, delirium, disability, and improved survival rates [8,9]. The primary goal of surgery is to restore the hip's anatomical and mechanical integrity, facilitating effective rehabilitation and early return to ambulation. Ferris et al. reported that patients who did not mobilize within one day after surgery had a 46% higher in-hospital mortality rate than those who mobilized early postoperatively [10]. Similarly, early mobilization has been linked to a 1.9-fold decrease in postoperative complications and a significant reduction in the incidence of delirium [11].

Several studies have demonstrated that early postoperative mobilization, defined as the ability to sit or stand out of bed shortly after surgery, is associated with improved gait outcomes [12-18]. Kuru et al., Goubar et al., and Su et al. reported better walking ability at 30 days postoperatively among patients who mobilized early [15-17]. Recognizing its importance, the National Hip Fracture Database includes "prompt mobilization" as a key performance indicator and defines early mobilization as the day after surgery [19]. However, the long-term effects of early mobilization, particularly regarding gait recovery one year after surgery, remain unclear. Previous studies have primarily assessed immediate postoperative outcomes, such as time to first mobilization and early postoperative walking ability, rather than sustained functional recovery. While these short-term outcomes are valuable indicators of early progress, their relationship with long-term gait function remains uncertain. Available studies have often been limited by relatively short follow-up periods and methodological inconsistencies, making it difficult to determine whether early mobilization leads to sustained improvements in functional independence. In addition, early mobilization practices vary significantly globally, with rates of 55-90% [20]. These differences may be influenced by multiple factors, including cultural attitudes toward rehabilitation, availability of healthcare resources, and institutional policies on postoperative care. Such variability underscores the importance of developing standardized mobilization protocols to enhance the comparability of study findings and improve global rehabilitation strategies.

This cohort study aimed to investigate the association between early mobilization on the first postoperative day and walking independence at one year postoperatively (primary outcome) in older adults after hip fracture surgery. Additionally, we examined gait recovery (secondary outcome), defined as the ability to walk using the same walking aid, or no aid, as before the injury.

## Materials and methods

Study design and participants

This prospective cohort study was conducted at Matsumoto City Hospital in Japan in accordance with the Strengthening the Reporting of Observational Studies in Epidemiology (STROBE) guidelines [21]. Data were collected from April 2018 to April 2020, with follow-up through April 2021. Mobility assessments were performed by trained physiotherapists involved in patient care; therefore, blinding was not possible. However, all assessments followed a standardized protocol to ensure consistency. The clinical team responsible for patient care conducted these measurements, which may have influenced mobilization practices. This limitation was acknowledged in interpreting the study results. Patients were included if they were able to walk independently indoors, regardless of the use of assistive devices (ambulatory), aged ≥65 years before the injury, had a femoral neck or trochanteric fracture, and were hospitalized for surgical treatment. Only patients who were permitted full weight bearing from the first postoperative day, regardless of the surgical method, were included in the study. The Institutional Ethics Committee of Matsumoto City Hospital approved the study on May 23, 2017. Informed consent was obtained from all participants. Study data, including objectives, criteria, and outcomes, were registered with UMIN-CTR (UMIN000054199).

Measurement

Patients with postoperative hip fractures were followed up prospectively to assess gait recovery. The patients were divided into two groups: the early mobilization (EM) group, comprising patients who were mobilized on the day after surgery, and the late mobilization (LM) group, including patients who were mobilized ≥2 days after surgery [6,16,19]. Postoperative mobilization was defined as the ability to sit or stand out of bed with or without help [6,19]. In addition to simple physical activities such as sitting or standing out of bed, a structured rehabilitation program was conducted for all patients after surgery. This rehabilitation program included physical therapy sessions focused on improving lower limb strength (e.g., quadriceps and hip abductor exercises), postural balance (e.g., weight-shifting and static balance training), and gait training (e.g., parallel bar exercises and overground walking with assistive devices as needed). The rehabilitation regimen was tailored to each patient’s needs, and generally involved daily sessions lasting 20 to 40 minutes, starting on the day after surgery or as soon as the patient was medically stable. Initially, passive or assisted exercises were implemented for patients with limited mobility, progressing to active resistance exercises and functional training. The program also emphasized weight-bearing activities as tolerated and encouraged independent walking as early as possible. The rehabilitation continued throughout the hospitalization period, aiming to maximize recovery and promote independent walking by the time of discharge from the hospital.

Demographic and background data were collected through interviews with patients and family members, as well as from medical records. We collected the following data from the included patients: 1) background data such as age, sex, body mass index, comorbidity diseases (cardiovascular, respiratory, kidney, and neurological diseases), and the Hasegawa Dementia Scale Revised (HDS-R) questionnaire (the HDS-R [22], as a measure of cognitive function, was assessed within one week postoperatively); 2) walking status before injury (walking without aids, with one-point cane, with walker, and with other aids); 3) place of residence before admission; and 4) medical data such as fracture type, surgical procedure, waiting days for surgery, complications [11] (deep vein thrombosis {DVT}, peroneal nerve palsy, infectious disease), and time to first mobilization after surgery (in days). In addition, the study collected data on several treatment outcomes, including the number of in-hospital days, walking status at discharge, walking functional independence measure (FIM) [23], destination, walking status at one year, walking FIM at one year, death, readmission, and re-fracture at one year postoperatively.

The main outcome was independent walking at discharge and one year postoperatively. We defined walking independently as a walking FIM ≥5, regardless of the walking aid used, following the approach outlined by Fu et al. [24]. FIM score ≥5 for walking indicates that the patient can with supervision or independently [23]. The secondary outcomes were gait recovery at discharge and one year postoperatively, defined by whether the patient had recovered to pre-injury walking status and walking FIM ≥5. Information regarding gait status was collected by telephone or through a questionnaire one year after surgery. We classified walking status based on the use of walking aids, similar to the approach used by Goubar et al. in their study on hip fracture recovery [16]. However, unlike previous studies, our evaluation was restricted to indoor ambulation only. Although we recognize the significance of home and community ambulation function, our focus on the use of indoor walking aids provides a clear and consistent measure of mobility that is particularly relevant to the daily living environment of older patients. By focusing on indoor ambulation, we aimed to capture the functional mobility that directly affects the patient's ability to perform essential daily activities within their living space.

The independent variables were EM and LM. The date of mobilization was defined as the date when the patient started mobilization, regardless of assistance. We adjusted for confounding factors that influence early postoperative mobilization based on previous studies [16,18]. These factors included age, pre-injury walking ability, cognitive decline, and fracture type. Cognitive impairment was defined as an HDS-R score of ≥21 [22].

The study sample size was determined to be 100 patients based on a previous study [2] that reported a 70% rate of independent walking one year after surgery and 10 events per covariate [25].

Statistical analysis

Continuous variables were presented as medians (interquartile range {IQR}), whereas categorical variables were expressed as numbers and percentages. The treatment outcomes between the EM and LM groups were compared using Pearson’s chi-square test, Fisher’s exact test for count data, and the Mann-Whitney U test.

Multivariate logistic regression analysis was performed to investigate the effect of early postoperative mobilization on independent walking one year postoperatively. The dependent variable in this analysis was the patient’s ability to walk independently one year postoperatively, whereas the independent variables were early and late postoperative mobilization. All confounding variables were synthesized into propensity scores, which were calculated using a model for predicting the postoperative mobilization timing. As the amount of missing data, including the loss to follow-up data, exceeded 5%, we performed multiple imputation and logistic regression analyses to account for the missing data [26]. To address missing data in the dataset, we performed multiple imputations using the “mice” package in R software (version 4.3.2, https://www.r-project.org), creating 20 complete datasets, using predictive mean matching for continuous variables and logistic regression for categorical variables. Afterward, logistic regression models were applied to each imputed dataset, and the results across the imputed datasets were integrated using Rubin’s rules [27].

For the sensitivity analysis, we examined the potential effect of unknown confounders on the relationship between postoperative early mobilization and independent walking by calculating E-values (https://www.evalue-calculator.com/) [28]. Furthermore, we conducted a sensitivity analysis using complete data to verify the robustness of the multiple imputation methods and assess potential biases introduced by missing data patterns. Statistical analyses were performed in R software (version 4.3.2, https://www.r-project.org). Statistical significance was set at P<0.05.

## Results

**Figure 1:**
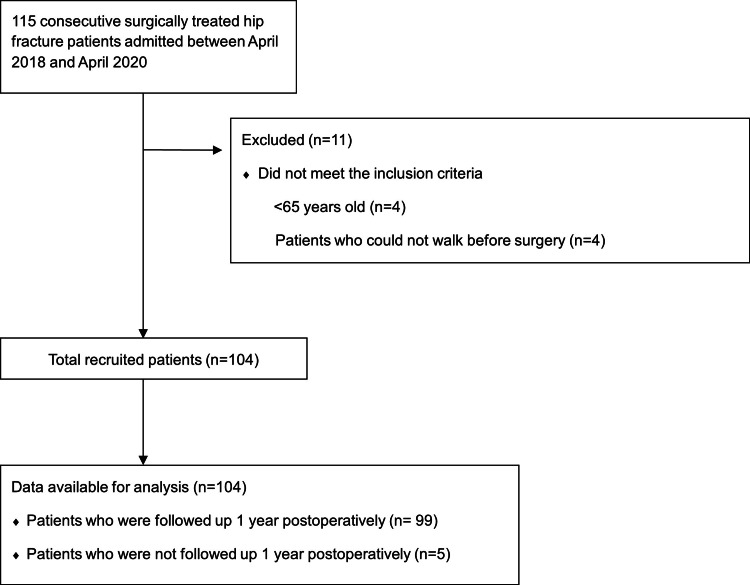
Flow diagram of patient recruitment.

In total, 104 patients were included in this study (Figure 1): 77 (74.0%) in the EM group and 24 (23.1%) in the LM group.

The median age of the patient was 86 years (IQR: 10 years). There were three cases (2.9%) of missing data for the first mobilization day postoperatively, and five patients (4.8%) were lost to follow-up one year postoperatively. The median ages (IQR) of the EM and LM groups were 86 (11.5) and 88 (5.5) years, respectively. Prior to the injury, 40 (52.6%) and 10 (41.7%) patients in the EM and LM groups, respectively, were able to ambulate without aids. Cognitive impairment was present in 27 (39.7%) and 12 (52.2%) patients in the EM and LM groups, respectively. The median hospital stay was 53 days (IQR: 31) in the EM group and 62.5 days (IQR: 30.5) in the LM group (Table 1).

**Table 1:** Patient demographic data. Data are presented as numbers (percentages) or medians (IQR). EM: early mobilization, LM: late mobilization, NMV: number of missing values, BMI: body mass index, DVT: deep vein thrombosis, IQR, interquartile range.

	All n = 104	EM group n = 77	LM group n = 24	NMV
Age (years)	86 (10)	86 (11.5)	88 (5.5)	2
Sex, n (%)				
Male	16 (15.4)	12 (15.6)	4 (16.1)	0
BMI (kg/m^2^)	20.0 (4.0)	20.2 (4.1)	19.9 (4.0)	3
Pre-fracture living situation, n (%)				0
-Own home	87 (83.7)	63 (81.8)	21 (87.5)	
-Nursing facility	17 (16.3)	14 (18.2)	3 (12.5)	
Pre-fracture walking status, n (%)				4
-Walking without aids	51 (49.5)	40 (52.6)	10 (41.7)	
-Walking with a one-point cane	22 (21.4)	15 (19.7)	6 (25.0)	
-Walking with a walker	30 (29.1)	21 (27.6)	8 (33.3)	
Cognitive impairment, n (%)	40 (42.6)	27 (39.7.)	12 (52.2)	13
Medical comorbidities, n (%)				
-Respiratory disease	9 (8.6)	8 (10.4)	0 (0)	0
-Cardiovascular disease	33 (31.7)	24 (31.2)	7 (29.2)	0
-Neurological disease	19 (18.3)	13 (16.9)	6 (25)	0
-Chronic kidney disease	8 (7.7)	6 (7.8)	2 (8.3)	0
-Diabetes	10 (9.6)	7 (9.1)	3 (12.5)	0
Fracture type, n (%)				2
-Femoral neck	50 (49.0)	40 (53.3)	10 (41.7)	
-Trochanteric	52 (51.0)	35 (46.7)	14 (58.3)	
Surgical treatment, n (%)				2
-Osteosynthesis	23 (22.6)	17 (22.7)	6 (25.0)	
-Artificial femoral head	79 (77.5)	58 (77.3)	18 (75.0)	
Serum albumin before surgery	3.7 (0.5)	3.7 (0.5)	3.6 (0.5)	5
Complication during hospitalization, n (%)				
-Peroneal nerve palsy	0 (0)	0 (0)	0 (0)	1
-DVT	0 (0)	0 (0)	0 (0)	1
-Infection	12 (11.5)	10 (13.0)	2 (8.3)	0
Waiting days for surgery	3.5 (3)	3 (3)	4 (1.5)	4
Time to first postoperative mobilization (days)	1 (0)	1 (0)	2 (1)	3
Hospital stay (days)	55 (34.3)	53 (31)	62.5 (30.5)	4
Destination after discharge, n (%)				0
-Own home	74 (71.2)	58 (75.3)	16 (66.7)	
-Nursing home	28 (26.9)	19 (24.7)	7 (29.2)	
-Death	2 (1.9)	0 (0)	1 (4.7)	

**Figure 2:**
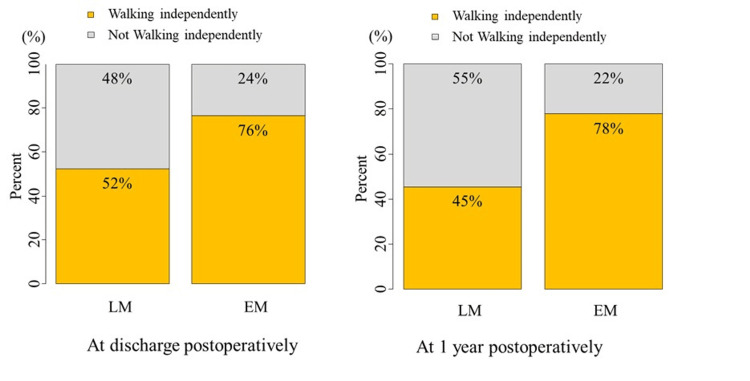
Early mobilization after surgery and walking independently. EM: early mobilization group, consisting of patients who were mobilized on the day after surgery, LM: late mobilization group, consisting of patients who were mobilized two days or more after surgery.

The number (%) of patients who were walking independently at discharge and one year postoperatively was 63 (60.6%) and 66 (63.5%), respectively. The EM group had a significantly higher percentage of patients who could walk independently at discharge and at 1 year postoperatively compared to the LM group (P = 0.03, 0.004, respectively) (Table 2, Figure 2). The number of patients who recovered to their pre-injury walking status at discharge and 1 year postoperatively was not significantly different between the two groups (P = 0.54, 0.08, respectively) (Table 2). There were no significant differences in death, rehospitalization, or re-fracture rates within 1 year between the two groups (P = 0.94, 0.53, and 0.74, respectively).

**Table 2:** Comparison of treatment outcomes between two groups. Data are presented as numbers (percentages). Test statistic (χ²): The chi-square value calculated for comparisons using χ² tests. * Fisher’s exact test was used where expected cell counts were <5. ** Chi-square test (χ²) was used for categorical variables. Statistical significance was set at p < 0.05. EM: early mobilization, LM: late mobilization, NMV: number of missing values, FIM: functional independence measure, N/A: not applicable.

	EM group n = 77	LM group n = 24	Test statistic (χ²)	P-value	NMV
Walking status at discharge, n (%)			N/A	0.02^*^	1
-Walking without aids	4 (5.3)	0 (0)			
-Walking with a one-point cane	31 (40.8)	3 (12.5)			
-Walking with a walker	33 (43.4)	16 (61.7)			
-Wheelchair	8 (10.5)	4 (16.7)			
-Death	0 (0)	1 (4.2)			
Walking FIM at discharge ≥5, n (%)	52 (76.5)	11 (52.4)	4.73	0.03^**^	0
Walking FIM 1-year after surgery ≥5, n (%)	56 (77.8)	10 (45.5)	8.47	0.004^**^	10
Recovery to pre-injury waling status at discharge, n (%)	24 (31.6)	6 (25)	0.38	0.54^**^	1
Recovery to pre-injury walking status 1 year after surgery, n (%)	35 (48.6)	6 (27.3)	3.04	0.08^**^	10
Mortality within 1 year, n (%)	3 (4)	1 (4.3)	N/A	0.94^*^	5
Readmission within 1 year, n (%)	14 (18.7)	3 (13)	2.39	0.53^**^	5
Re-fracture within 1 year, n (%)	5 (6.7)	2 (18.7)	2.36	0.12^**^	5

After adjusting for confounders, multivariate logistic regression analysis showed that EM within one day of surgery was associated with independent walking one year postoperatively, with an odds ratio (OR) of 3.79 (95% confidence interval {CI}: 1.30-11.06) (Table 3). However, no significant association was found between early postoperative mobilization and recovery of pre-injury walking status one year postoperatively (Table 4).

**Table 3:** Odds ratios for walking independence one year postoperatively, associated with early postoperative mobilization in older patients after hip fracture surgery. Data are presented as N (%). Statistical significance was set at p < 0.05. CI: confidence interval, ref: reference. * Applying the propensity score calculated using a model for predicting the postoperative mobilization timing, whether early or late mobilization, considering the confounding variables age, preoperative walking ability, cognitive impairment, and fracture type.

Variables	Patients, n (%)	Walking independence rate, n (%)	Adjusted odds ratio*	95% CI		p-value	Adjusted odds ratio*	95% CI		p-value
				Lower	Upper			Lower	Upper	
At 1 year postoperatively	Completed data (n=94)						Multiple imputation data			
Late mobilization >1 day from surgery to mobilization	22 (23.4)	10 (45.5)	1.00 (ref)				1.00 (ref)			
Early mobilization ≤1 day from surgery to mobilization	72 (76.6)	56 (77.8)	3.28	1.07	10.02	0.037	3.79	1.30	11.06	0.01

**Table 4:** Odds ratios for recovery of pre-injury walking status one year postoperatively, associated with early postoperative mobilization in older patients after hip fracture surgery. Data are presented as N (%). Statistical significance was set at p < 0.05. CI: confidence interval, ref: reference. * Applying the propensity score calculated using a model for predicting the postoperative mobilization timing, whether early or late mobilization, considering the confounding variables: age, preoperative walking ability, cognitive impairment, and fracture type.

Variables	Patients, n (%)	Recovery of pre-injury walking status, n (%)	Adjusted odds ratio*	95% CI		p-value	Adjusted odds ratio*	95% CI		p-value
				Lower	Upper			Lower	Upper	
At 1 year postoperatively	Completed data (n=94)						Multiple imputation data			
Late mobilization >1 day from surgery to mobilization	22 (23.4)	6 (27.3)	1.00 (ref)				1.00 (ref)			
Early mobilization ≤1 days from surgery to mobilization	72 (76.6)	35 (48.6)	1.65	0.53	5.51	0.40	1.93	0.61	6.12	0.22

Sensitivity analysis yielded comparable results for models that used complete data and those that accounted for multiple assignments of missing values. Subsequently, to evaluate the influence of potential unmeasured confounders on the relationship between postoperative early mobilization and independent walking at one year postoperatively, we calculated the E-value for the point estimate (3.3) and the CI (1.54).

## Discussion

Our findings provide evidence that early mobilization one day after hip fracture surgery is significantly associated with independent walking at one year postoperatively. The percentage of participants in our study who walked independently after one year was 63.5% (66 of 104 participants), which was similar to the findings of Fu et al. who evaluated independent walking using the same criteria (FIM score ≥5) [24]. Early mobilization is associated with gait recovery at 30 days and two months postoperatively in patients with hip fractures [15-17]. Furthermore, we provide new evidence that early mobilization is associated with independent walking one year after surgery. A previous study reported that mobility at discharge was associated with ambulatory ability one year postoperatively [2]. Some previous studies have indicated that early postoperative mobility improves walking ability at one or two months [15-17]. Considering the results of previous studies [15-17], reacquiring the ability to walk at an early stage (30 days or two months) via early mobilization may affect walking independently one year later. In our study, no significant difference in postoperative complications was observed between the early mobilization and usual care groups. However, early mobilization may have contributed to improved gait function at one year by increasing opportunities for standing and walking exercises, thereby facilitating muscle strength recovery. The precise mechanisms underlying this effect remain unclear, highlighting the need for further research to better understand how early mobilization supports long-term functional outcomes.

In contrast to the findings of Goubar et al., we found no association between early mobilization and recovery to pre-injury walking status [16]. This lack of association could be affected by several factors that were not measured in this study, including the individualization of the care provided by the rehabilitation staff. The variability in the prescription of walking aids probably reflects an approach to personalized patient care rather than a deficiency in standardization. However, this raises important questions about the factors that affect walking aid decisions and how these decisions impact recovery. Future studies should explore the role of walking aid prescription in rehabilitation outcomes to better understand these dynamics.

According to global data, the proportion of patients mobilized early is 55-90% in several countries [20], indicating significant variations in early mobilization practices worldwide. These variations may reflect differences in healthcare resources, staffing levels, or cultural approaches to patient care. Based on the results of previous studies and those of the present study [6,12,14,15], we suggest that clinicians should promote early mobilization postoperatively in the absence of a contraindicated condition that makes mobilization difficult.

This study has several limitations. First, 9.6% (10 of 104 participants) had missing values regarding outcomes at one year, and 12.5% (13 of 104 participants) had missing values regarding cognitive function tests. Therefore, we assumed that the defect pattern was missing at random and performed multiple imputations. In addition, we performed a sensitivity analysis of the complete data and obtained results similar to those of the multiple imputation method, confirming the robustness of the statistical method used in this study.

Second, the lack of blinding introduces the potential for observer bias, as the physical therapist conducting the mobility assessments was also involved in patient care. Although a standardized protocol was used to ensure consistency, this does not completely eliminate the possibility of bias. Future studies should consider independent assessors or objective measurement tools to enhance the reliability of mobility evaluations.

The barriers to early mobilization require further investigation. Although our study did not aim to measure these barriers directly, previous studies have identified factors such as patient confusion, pain, and hypotension as significant challenges [29]. These barriers are critical to understanding and improving early mobilization strategies. Future studies should focus on identifying and addressing these barriers within clinical settings to improve postoperative recovery protocols.

Finally, this study included a single-center cohort with a relatively small sample size, which may limit the generalizability of the findings. Additionally, the exclusion of non-ambulatory patients and those under 65 years of age further restricts the applicability of our results to younger individuals and patients with different functional backgrounds.

Moreover, while the E-value approach provides an estimate of the minimum strength of association that an unmeasured confounder would need to explain away the observed results, it does not directly quantify or adjust for these confounders. Therefore, residual confounding cannot be entirely ruled out. Future studies with more comprehensive data on potential confounders are needed to further validate our findings.

This study also has several strengths. First, the follow-up period after hip fracture surgery was one year. Although most previous studies describe a follow-up of two months, this study is considered novel because it monitored the prognosis of walking at one year postoperatively [12,16,17]. Second, we used outcomes and confounding factors that are commonly used in clinical practice. After adjusting for confounding factors assumed in previous studies, we examined the effects of early mobilization after surgery on walking independently.

## Conclusions

This study found that early postoperative mobilization (EM) is significantly associated with a higher likelihood of independent walking one year after hip fracture surgery in older patients. Patients who mobilized early were nearly four times more likely to achieve independent ambulation, highlighting the importance of incorporating EM into standard postoperative care.

Future research should address barriers to EM and validate these findings through larger, multicenter studies to guide evidence-based rehabilitation practices.
